# Ruptured Iliac Arteriovenous Fistula Presenting With Thigh Pain and Swelling: Case Report

**DOI:** 10.3389/fsurg.2022.834071

**Published:** 2022-03-16

**Authors:** Claire Morton, Kendal M. Endicott, Annalise Penikis, Shahab Toursavadkohi, Michael R. Hall

**Affiliations:** ^1^Division of Vascular Surgery, Department of Surgery, University of Maryland, Baltimore, MD, United States; ^2^Inova Heart and Vascular Institute, Inova Health Systems, Falls Church, VA, United States

**Keywords:** case report, AV fistula, high output heart failure, endovascular, common iliac aneurysm

## Abstract

The presentation of abdominal arteriovenous fistulas is classically described as a triad of a pulsatile abdominal mass with a bruit, high-output heart failure, and regional venous hypertension with primarily open operative therapy. In the following case, we present the treatment of a patient who arrived with acute right heart failure and renal failure due to an arteriovenous fistula and who was successfully treated with endovascular repair.

## Introduction

Abdominal arteriovenous fistulas are rare and most commonly caused by a spontaneous rupture of an atherosclerotic abdominal aortic aneurysm into the adjacent inferior vena cava (ICV). While an uncommon presentation of abdominal aortic rupture, the classically described clinical presentation is a pulsatile abdominal mass with an abdominal bruit, high-output congestive cardiac failure, and regional venous hypertension (1). The physiologic consequences of the abdominal arteriovenous fistulas (AVF) are determined by the size of the fistula and subsequent flow volume, as well as the acuity of fistula formation (2).

Prompt diagnosis, as well as carefully planned surgical and anesthetic management, are critical to patient outcomes. While open surgery was once the only option for repair, endovascular therapy has emerged as a promising alternative, particularly in a patient with significant physiologic derangements. In addition, intraoperative anesthesia management of hemodynamic volatility is crucial.

In this case report, we describe the unique presentation and perioperative management of a patient with a ruptured, right common iliac artery aneurysm with fistulous connection to the right common iliac vein resulting in high output cardiac failure and acute renal failure successfully treated with endovascular stent grafting.

## Case Description

A 58-year-old man with a past medical history of hypertension, gastroesophageal reflux, and tobacco abuse presented to an outside hospital emergency department with right thigh pain and swelling. He was diagnosed based on the exam alone with a deep venous thrombosis (as the ultrasound was not available) and discharged on therapeutic anticoagulation with Lovenox.

**Figure 1:**
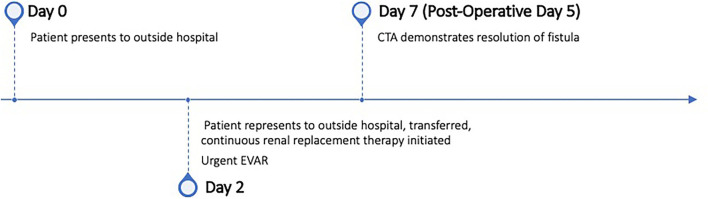
Care timeline.

The patient returned 2 days later, reporting abdominal pain as well as new-onset chest pain and mild shortness of breath. Serologies revealed a newly elevated creatinine at 9.3 mg/dl (baseline 1.1 mg/dl) with rising troponins up to 3.7 ng/ml. ECG revealed atrial fibrillation with ST-segment depressions. Duplex ultrasound revealed no deep vein thrombosis (DVT) but impeded venous flow throughout. CT scan with oral contrast was obtained to evaluate his abdominal pain. This imaging demonstrated bilobed aneurysmal dilation of the right common iliac artery (CIA). A study with intravenous contrast was deferred due to his renal dysfunction. The patient was subsequently transferred to our institution for further evaluation (Figure 1).

**Figure 2:**
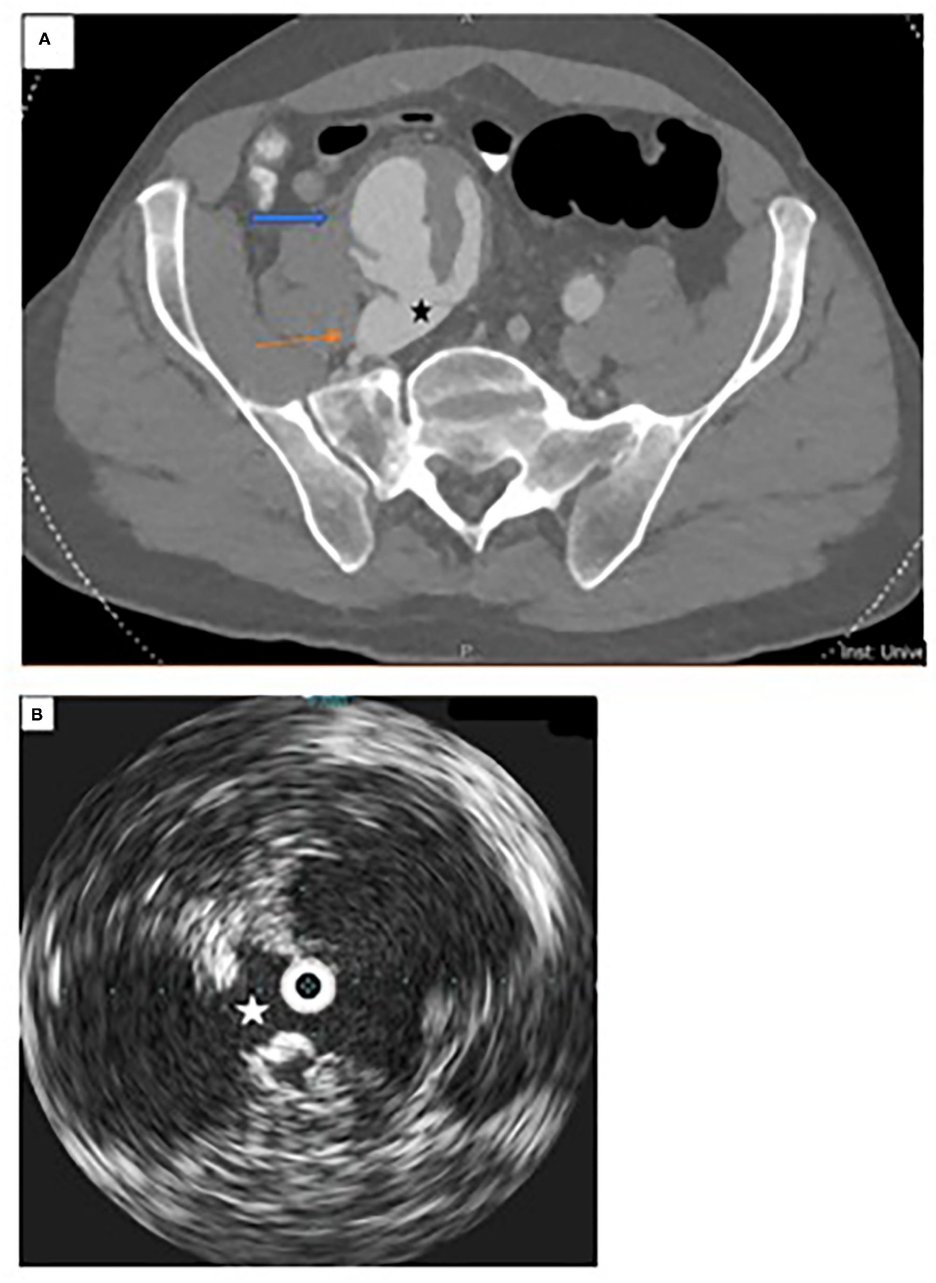
Additional imaging of fistula. **(A)** CT angiogram demonstrating arterial-venous fistula between the common iliac artery (blue arrow) and common iliac vein (orange arrow). **(B)** Intravascular ultrasound demonstrating arterial-venous fistula between the common iliac artery and common iliac vein.

Upon arrival, the patient was diaphoretic, dyspneic, and hypotensive, requiring high dose vasopressor support. On exam, his abdomen was tender with a pulsatile mass in the right lower quadrant without signs of peritonitis. The right leg demonstrated severe edema with a palpable thrill in the groin with no discernable Doppler signals below the common femoral artery. Pulses in the left leg were palpable. The patient was immediately placed on continuous venovenous hemofiltration due to renal failure. He subsequently underwent a CT angiogram (CTA) demonstrating a right CIA aneurysm, a maximal diameter of 5.8 cm, with arteriovenous fistula to the right common iliac vein (Figure 2). The infrarenal aorta measured 3.7 cm in diameter with a normal neck diameter of ~24 mm in length.

The deteriorating clinical status of the patient was attributed to acute heart failure from the high-volume arteriovenous fistula. He was taken to the operating room for urgent endovascular aortic repair (EVAR). The patient was intubated without event; however, he also required increasing vasopressor support. Following percutaneous large bore access of the bilateral common femoral arteries, a 12 French sheath was also placed in the right common femoral vein for temporary balloon occlusion of the IVC in the event of cardiovascular collapse.

Initial arteriogram demonstrated early iliac venous filling with opacification of the IVC consistent with CT imaging. No opacification of the right iliac system distal to the AVF was seen. Intravascular ultrasound (IVUS) demonstrated the large arteriovenous fistula between the right CIA and the right common iliac vein (CIV) (Figure 2). While coil embolization of the right hypogastric artery had initially been planned for adequate seal zone of the right iliac limb, anatomy and stenosis of the origin of the right hypogastric artery prevented expeditious cannulation. Given the tentative clinical status of the patient, the decision was made to proceed with EVAR and coverage of the right hypogastric artery.

In preparation for possible massive hemodynamic lability, an occlusive balloon was placed *via* the venous sheath to slowly and temporarily occlude the fistula. Occlusion of the fistula increased the systolic pressure of the patient by 80 mmHg and allowed the anesthesia team to anticipate the patient's hemodynamic response to device deployment across the fistula. In addition, the balloon was retained in the CIV in the event that endovascular intervention was unsuccessful and urgent open repair was deemed necessary.

The main body of a Gore Excluder endoprosthesis 26 × 14 × 14 mm (W. L. Gore & Associates Inc, Flagstaff, Arizona) was deployed with subsequent gate cannulation and deployment of the left iliac limb. Upon deployment of the right iliac limb (Gore Excluder 12 × 12 × 10 mm, Gore Excluder 16 × 14 × 7 mm, Gore Excluder 18 × 12 mm, and Gore Excluder 18 × 10 mm) across the fistulous connection, the patient became acutely hypertensive and was immediately weaned from vasopressors. While previously anuric, the patient immediately began producing urine with a subsequent output of 250 cc in the next hour with greater than 100 cc/h thereafter.

**Figure 3:**
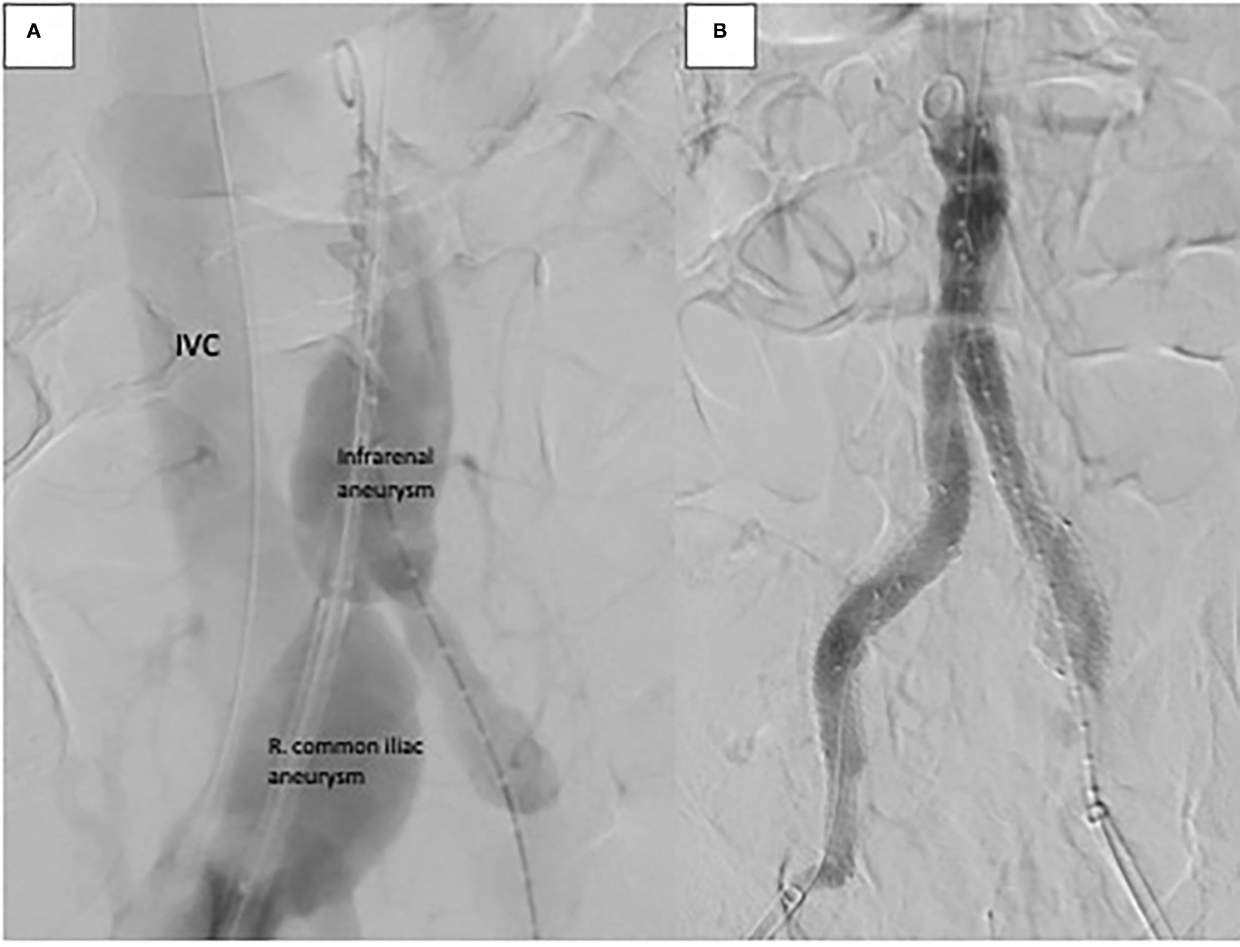
Intra-operative angiography. **(A)** Aortogram demonstrating infrarenal and right common iliac aneurysm with rapid filling of inferior vena cava (IVC) suggesting aortovenous fistula. **(B)** Aortogram after endovascular repair demonstrating exclusion of aortoiliac aneurysms and fistula.

The final arteriogram demonstrated no significant endoleak and no filling of the venous system (Figure 3). Venogram demonstrated filling of the vena cava with no extravasation or filling of the iliac artery aneurysm. The patient was extubated without event and taken to the cardiac intensive care unit for further management. Post-operatively, the patient required no further hemofiltration. Pulses in the right leg returned with cessation of chest pain. Creatinine rapidly corrected and was in the normal range by day five of post-operation. Post-operative echocardiogram (ECHO) demonstrated normal LV size with moderately decreased systolic function with an ejection fraction of 40%. Wall motion abnormalities detected on ECHO suggested a left circumflex branch occlusion with lateral wall hypokinesis, likely a sequela of his presenting pathology.

Repeat CTA on day five of post-operation demonstrated closure of the arteriovenous fistula with a small, expected, type II endoleak from the right hypogastric artery. Given the size of the fistula, we attempted to embolize this hypogastric artery *via* a femoral venous approach. We were able to access the right common iliac artery aneurysm sac from this approach but again could not cannulate the right hypogastric artery given the ostial stenosis. The decision was made for no further intervention and serial surveillance imaging.

**Figure 4:**
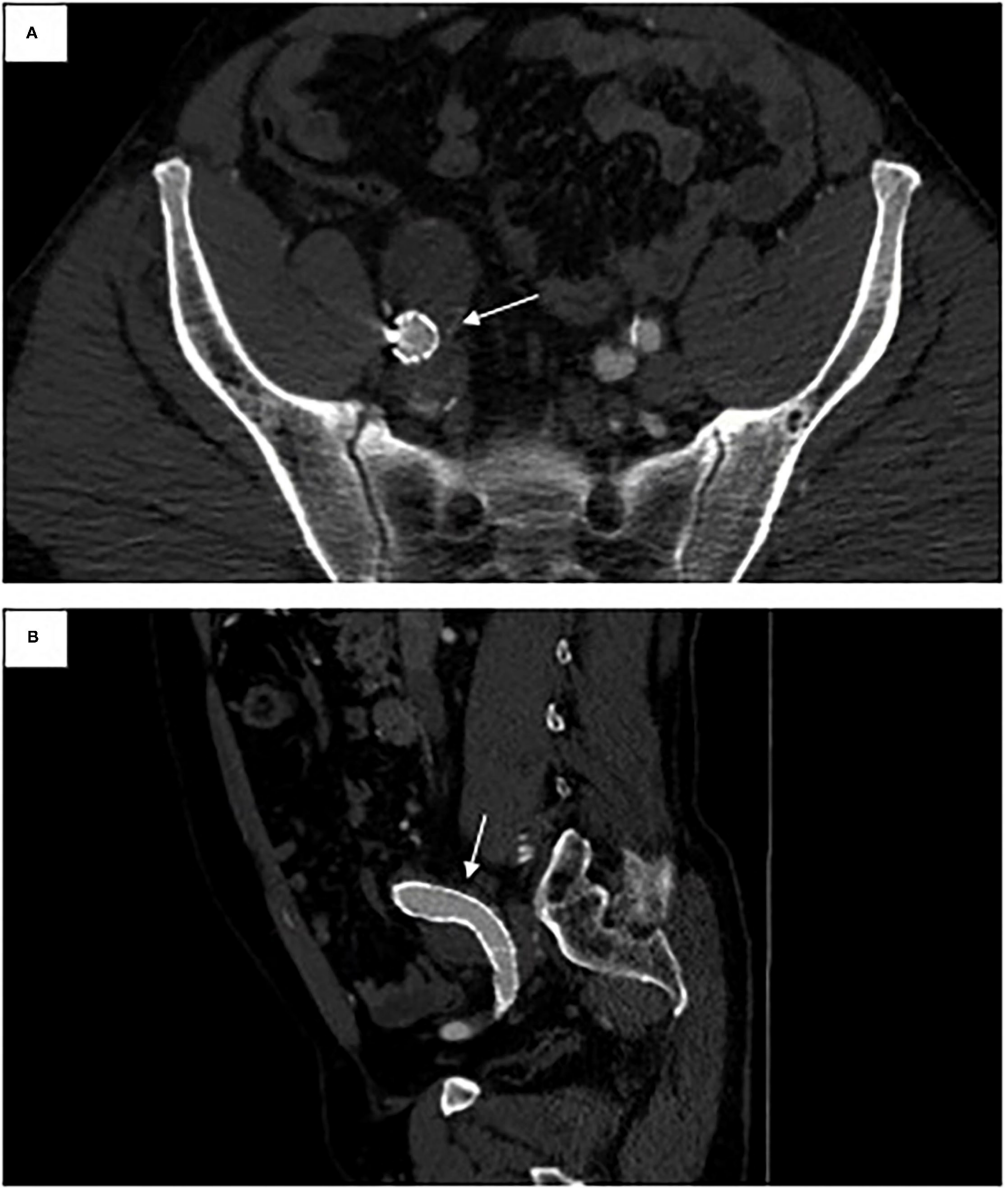
One-year follow-up CT angiogram. **(A)** Axial view of right iliac limb in previous fistula location. **(B)** Sagittal view of right iliac limb in previous fistula location.

Repeat imaging at 1-, 6-, and 12-month follow-up intervals initially demonstrated a type II endoleak (likely from a patent inferior mesenteric artery) with resolution on serial imaging. The aortic and right iliac aneurysms both demonstrated a decrease in size (Figure 4). At 1 year follow-up, the infrarenal aortic sac measured 32 mm (from 37 mm). The bilobed right CIA decreased to a maximum diameter of 32 mm (from the initial maximum diameter of 58 mm). At 1-year follow-up, the patient continues to do well overall with no sequelae from the acute renal failure or myocardial infarction.

## Discussion

The primary development of abdominal AVFs from abdominal aortic aneurysms is thought to be due to gradual erosion of the aortic wall. The pressure and tension from large arterial aneurysms create an inflammatory interface with the adjacent vein. This may cause eventual necrosis of the aortic wall and degradation of the arterial wall, resulting in fistula formation. The physiologic consequences can present immediately with life-threatening, acute onset heart failure or less commonly may be detected in a more chronic state of high output cardiac failure.

In the acute setting, the formation of the AVF results in an acute drop in systemic vascular resistance (SVR) as blood is shunted into the lower resistance venous system. A large AVF causes the central venous pressure to rise with a decrease in mean arterial pressure. Compensatory mechanisms increase venous return and heart rate to maintain cardiac output as well as cerebral and coronary perfusion. SVR increases secondary to direct sympathetic effects and stimulates renal renin secretion. The increased venous pressure creates a post-obstructive outflow issue for the kidneys, constituting the first component of kidney injury. In addition, the decrease in systemic blood flow that results from the shunting leads to decreased renal perfusion. (2) Ultimately, as was the case for this patient, the acute combination of decreased inflow with obstructed outflow causes rapid renal failure. In a presentation mimicking cardiorenal syndrome, in the case of a massive fistula, compensatory mechanisms are overwhelmed with subsequent signs of acute heart failure and renal impairment (3). Our patient demonstrated a dramatic presentation of this physiology as well as an equally dramatic improvement with the repair. In this case, when the fistula was occluded, and normal perfusion patterns resumed, the patient's kidneys were able to recover. The occlusion of the fistula prevented excess blood flow into the venous system, thereby reducing the outflow obstruction, and restoring the arterial inflow to the renal system.

The study of Albalate et al. (4) reports a general under-recognition and reporting of the associated acute renal failure (ARF). A recent Medline review additionally failed to list renal failure as a component of the initial presentation. Orion et al. (5) also do not discuss ARF as a component of the presentation. Nonetheless, the review conducted by Antoniou et al. (6) regarding cases treated with endovascular stent-grafts cites several cases of ARF, which also site “central cyanosis” and shock as a presenting feature. These observations as well as the extreme hemodynamic derangements in our case presentation support the notion that the size of the AVF, as well as the acuity of AVF formation, may play a role in the development of ARF. The immediate and robust return of urine output following treatment speaks to the immediate reversal of physiologic derangement upon repair.

Given our patient's tenuous hemodynamic state, we chose to obtain percutaneous common femoral venous access prior to induction of anesthesia and coverage of the fistula to facilitate caval balloon occlusion should the patient's hemodynamics worsen at any time. While the patient's hemodynamics remained stable throughout induction of anesthesia, we simulated the hemodynamic changes of AVF occlusion during placement of the endograft by performing caval balloon occlusion. This allowed the anesthesia team to prepare for the hemodynamic changes that would rapidly occur once the endograft excluded the AVF. We found this strategy beneficial to facilitate continuous communication with the anesthesia team in addition to ensuring the prevention of severe rebound hypertension following the closure of the fistula. Caval balloon occlusion may also be an effective strategy in open repair depending on the location of the AVF.

Our decision for endovascular repair is consistent with trends in the literature. While a recent review questioned endovascular therapy as definitive therapy due to the long-term persistence of endoleak in up to 50% of patients, that review also acknowledged the benefit of EVAR in the case of hemodynamic instability (5). While we ultimately aborted coil embolization of the right hypogastric artery, the anatomy was overall favorable for an endovascular repair, and follow-up demonstrates regression of the aneurysm sac with no current endoleak identified.

Overall, this case report describes a dramatic presentation of an abdominal AVF repaired with EVAR, focusing on the role of temporary balloon occlusion to aid in anesthetic intraoperative support. In addition, our case highlights the rapid correction of physiologic derangements associated with surgical correction as well as the benefit of endovascular repair.

## Data Availability Statement

The original contributions presented in the study are included in the article/supplementary material, further inquiries can be directed to the corresponding author.

## Ethics Statement

Ethical review and approval was not required for the study on human participants in accordance with the local legislation and institutional requirements. The patients/participants provided their written informed consent to participate in this study. Written informed consent was obtained from the individual(s) for the publication of any potentially identifiable images or data included in this article.

## Author Contributions

All authors listed have made a substantial, direct, and intellectual contribution to the work and approved it for publication.

## Conflict of Interest

The authors declare that the research was conducted in the absence of any commercial or financial relationships that could be construed as a potential conflict of interest.

## Publisher's Note

All claims expressed in this article are solely those of the authors and do not necessarily represent those of their affiliated organizations, or those of the publisher, the editors and the reviewers. Any product that may be evaluated in this article, or claim that may be made by its manufacturer, is not guaranteed or endorsed by the publisher.
